# Crystal structure of di­benzyl­ammonium hydrogen (4-amino­phen­yl)arsonate monohydrate

**DOI:** 10.1107/S205698902300837X

**Published:** 2023-10-05

**Authors:** Bocar Traoré, Waly Diallo, Mamadou Sidibé, Libasse Diop, Laurent Plasseraud, Hélène Cattey

**Affiliations:** a Laboratoire de Chimie Minérale et Analytique (LACHIMIA), Département de Chimie, Faculté des Sciences et Techniques, Université Cheikh Anta Diop, Dakar, Senegal; b ICMUB UMR 6302, Université de Bourgogne (UB), Faculté des Sciences, 9 avenue Alain Savary, 21000 DIJON, France; Universidad de Los Andes Mérida, Venezuela

**Keywords:** crystal structure, group 15 - pnictogen elements, organic salt, phenyl­arsonic derivatives, hydrogen bonds, infinite chain

## Abstract

The title salt, C_14_H_16_N^+^·C_6_H_7_AsNO_3_^−^·H_2_O or [(C_6_H_5_CH_2_)_2_NH_2_][H_2_NC_6_H_4_As(OH)O_2_]·H_2_O, (**I**), was synthesized by mixing an aqueous solution of (4-amino­phenyl)­arsonic acid with an ethano­lic solution of di­benzyl­amine at room temperature. Compound **I** crystallizes in the monoclinic *P*2_1_/c space group. The three components forming **I** are linked *via* N—H⋯O and O—H⋯O inter­molecular hydrogen bonds, resulting in the propagation of an infinite zigzag chain. Additional weak inter­actions between neighbouring chains, such as π–π and N—H⋯O contacts, involving phenyl rings, –NH_2_ and –As(OH)O_3_ functions, and H_2_O, respectively, lead to a three-dimensional network.

## Abstract 2

The title salt consists of three components, comprising one di­benzyl­ammonium cation, [(C_6_H_5_CH_2_)_2_NH_2_]^+^, one hydrogen (4-amino­phen­yl)arsonate anion, [H_2_NC_6_H_4_As(OH)O_2_]^−^, and one mol­ecule of water. In the crystal, these components are organized in infinite zigzag chains *via* inter­molecular hydrogen bonds. Weak inter­actions between the chains lead to a three-dimensional network.

## 1. Chemical context

Organoarsenic compounds have been known for a long time and sparked great inter­est when they were discovered. Tetra­methyl­diarsine (Me_2_As-AsMe_2_), commonly known as *Cacod­yl*, was isolated in the middle of the 18^th^ century by Cadet de Glaussicourt (Garje & Jain, 1999 ▸). During the next century, in 1859, Antoine Béchamp reported the synthesis of *p*-arsanilic acid sodium salt (named *Atox­yl*) by reacting aniline with arsenic acid. This compound was employed for pharmaceutical applications, in particular against trypanosomal infection. Subsequently, in the early 20th century, Paul Ehrlich was inspired by this work to develop a new organoarsenic derivative, called *Arsphenamine* or *Salvarsan* (Ehrlich & Bertheim, 1907 ▸). This mol­ecule has proved particularly effective in the treatment of syphilis and sleeping sickness (African Trypanosomiasis) and is considered as being the first chemotherapeutic agent (Williams, 2009 ▸). The use of organo­arsenicals as medicines was subsequently abandoned in favour of penicillin, as they were found to be highly toxic to humans, causing significant side effects (including blindness). However, they have continued to be used, until recently, as feed additives and veterinary drugs, particularly in the livestock and poultry breeding industry, but with serious negative effects on the environment. Soil and groundwater contamination resulting from the excessive use of aromatic organoarsenic compounds is now a major environmental concern (Fei *et al.*, 2018 ▸). Current investigations involving academics focus on improving analytical detection (Depalma *et al.*, 2008 ▸; Yang *et al.*, 2018 ▸) and remediation methods (Jun *et al.*, 2015 ▸; Chen *et al.*, 2022 ▸).

From a structural point of view, the crystal structure of phenyl­arsonic acid was first solved in the early 1960s (refcode ARSACP: Shimada, 1960 ▸). Since then, the X-ray structure for the zwitterionic form of *p*-arsanilic acid (*p*-ammonio­phenyl­arsonate) has been determined (CUDSEZ: Shimada, 1961 ▸; CUDSEZ01: Nuttall & Hunter, 1996 ▸) as well as of the hydrated ammonium and sodium salt hydrates of 4-amino­phenyl­arsonic acid (KOKWOY, KOKWUE: Smith & Wermuth, 2014 ▸). We report herein the structure of a new salt of 4-amino­phenyl­arsonate, isolated from a mixture of (4-amino­phen­yl)arsonic acid and di­benzyl­amine and characterized as di­benzyl­ammonium hydrogen (4-amino­phen­yl)arsonate monohydrate, [(C_6_H_5_CH_2_)_2_NH_2_][H_2_NC_6_H_4_As(OH)O_2_]·H_2_O (**I**). 
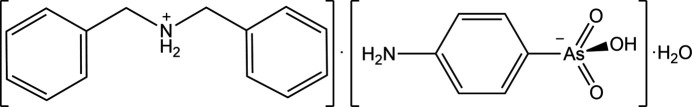


## 2. Structural commentary

**Figure 1:**
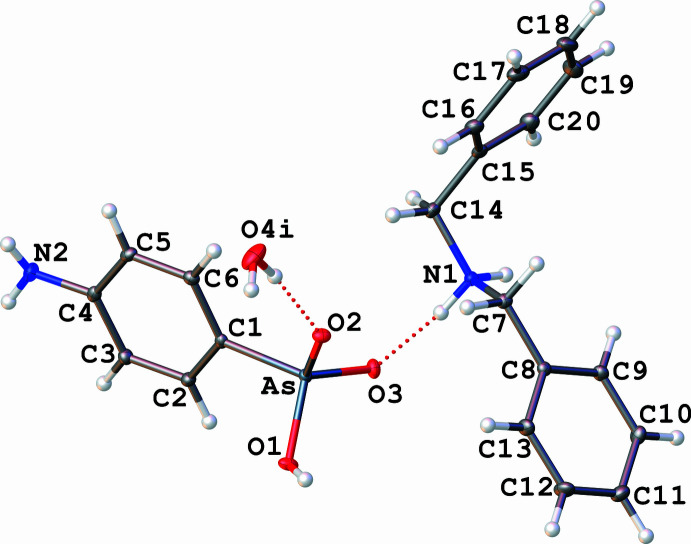
The mol­ecular structure of **I** with displacement ellipsoids at the 30% probability level.The water mol­ecule was found to be disordered over two positions, the minor part was omitted and the major part is represented with the following symmetry code: (i): −1 + *x*, *y*, *z*. Dotted lines indicate hydrogen bonds.

The asymmetric unit of the title salt, which is depicted in Fig. 1 ▸, comprises one di­benzyl­ammonium cation [(C_6_H_5_CH_2_)_2_NH_2_]^+^, one hydrogen (4-amino­phen­yl)arsonate anion [H_2_NC_6_H_5_As(OH)O_2_]^−^ and one water mol­ecule of solvation. The three components of **I** are linked together through inter­molecular N—H⋯O and O—H⋯O hydrogen bonds. The As atom of the anion is bonded to three O atoms and one carbon atom of the phenyl ring, describing a slightly distorted tetra­hedral geometry [O1—As—C1 = 103.71 (6)°, O2—As—C1 = 110.47 (6)°, O3—As—C1 = 111.73 (6)°, O2—As—O1 = 110.71 (5)°, O3—As—O1 = 108.46 (5)°, O3—As—O2 = 111.48 (5)°]. The As—O bonds exhibit two distinct lengths: As—O1 = 1.7267 (10) Å, and As—O2 = 1.6730 (10) Å and As—O3 =1.6699 (10) Å, which can be considered to be identical. The As—O1 distance is consistent with the presence of a hydroxyl group (Yang *et al.*, 2002 ▸), while the As—O2 and As—O3 distances, which are shorter, reflect rather a double-bond character. In the literature, based on a comparison of structural examples, the average length of the As—O bond is defined as 1.77 Å and that of the As=O bond as 1.67 Å (Nuttall & Hunter, 1996 ▸). The nature of the As=O2 and As=O3 double bonds implies that the negative charge is delocalized on the arsonate. The three oxygen atoms of the arsonate function are engaged in hydrogen bonding, the O1 and O2 atoms being linked head-to-tail [O1—H⋯O2^iv^, *D*⋯*A* = 2.5444 (15) Å; symmetry code: (iv) −*x*, −*y* + 1, −*z* + 1, Table 1 ▸]. The length of the As—C1 bond [1.8955 (13) Å] is within the range of values measured for related compounds such as ammonium 4-nitro­phenyl­arsonate (Yang *et al.*, 2002 ▸) and guanidinium phenyl­arsonate (Smith & Wermuth, 2010 ▸). An amino group is positioned on the phenyl ring in the *para* position to the arsonate function. Both functional groups are contained in the plane of the phenyl ring. The negative charge of [H_2_NC_6_H_4_As(OH)O_2_]^−^ is compensated by the presence of one di­benzyl­ammonium cation, [(C_6_H_5_CH_2_)_2_NH_2_]^+^, whose NH_2_^+^ group is hydrogen bonded to the oxygen atom O3 of the arsonate function [N1—H1*A*⋯O3, *D*⋯*A* = 2.6842 (16) Å, N1—H1*B*⋯O3^iii^, *D*⋯*A* = 2.7260 (15) Å; symmetry code: (iii) −*x* + 1, −*y* + 1, −*z* + 1]. Moreover, the di­benzyl­ammonium cation shows a *syn*–*anti* conformation, displaying C—C—N—C torsion angles of 57.65 (16)° and −178.14 (11)°, which are in the range of previous examples of X-ray structures involving [(C_6_H_5_CH_2_)_2_NH_2_]^+^ (Trivedi & Dastidar, 2006 ▸). A water mol­ecule (co-solvent of the reaction) participates in a hydrogen-bond inter­action with the oxygen atom O2 of –As(OH)O_2_^−^ [O4—H4*A*⋯O2^V^, *D*⋯*A* = 2.8074 (18) Å; symmetry code: (v) 1 + *x*, *y*, *z*] completes the composition of salt **I**. From a spectroscopic point of view, the infrared spectrum of **I** (ATR mode) highlights ν(As—C) and ν(As—O) absorption bands, which are characteristic of the arsonate function (Cowen *et al.*, 2008 ▸), at 1096 cm^−1^ and between 925–690 cm^−1^, respectively. The percentages of C, H, N and O determined by elemental analysis support the chemical composition of **I**, but show that the salt is partially dehydrated (see the *Synthesis and crystallization* section).

## 3. Supra­molecular features

**Figure 2:**
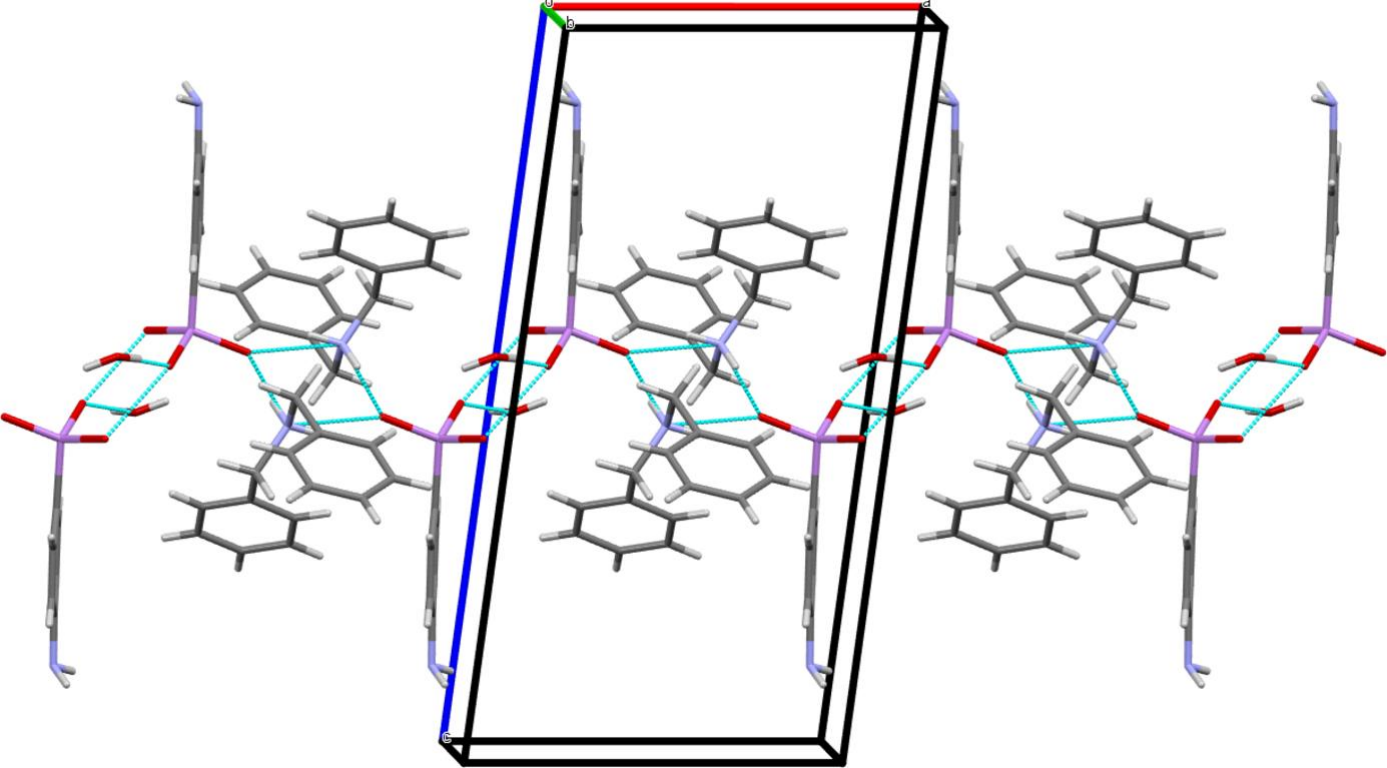
Mercury representation (Macrae *et al.*, 2020 ▸; colour code: C = grey, N = blue, O = red, As = pink, H = white] of the infinite chain structure of **I** propagating along the *a*-axis direction *via* hydrogen bonds (dotted cyan lines).

**Figure 3:**
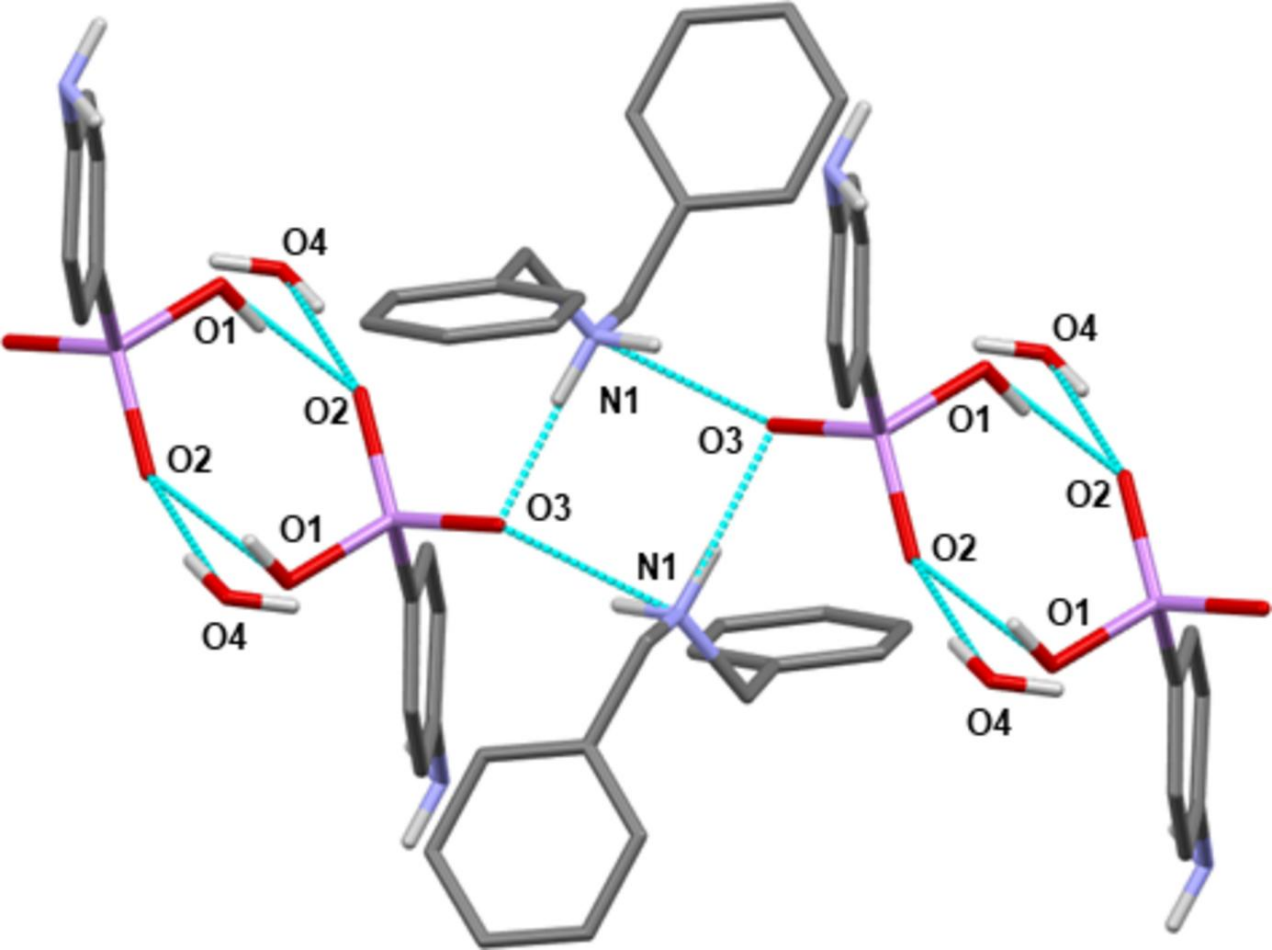
Mercury representation (Macrae *et al.*, 2020 ▸; colour code: C = grey, N = blue, O = red, As = pink, H = white) highlighting the hydrogen-bonding network (cyan dotted lines) involving the components of **I** (the benzyl H atoms have been omitted for clarity).

**Figure 4:**
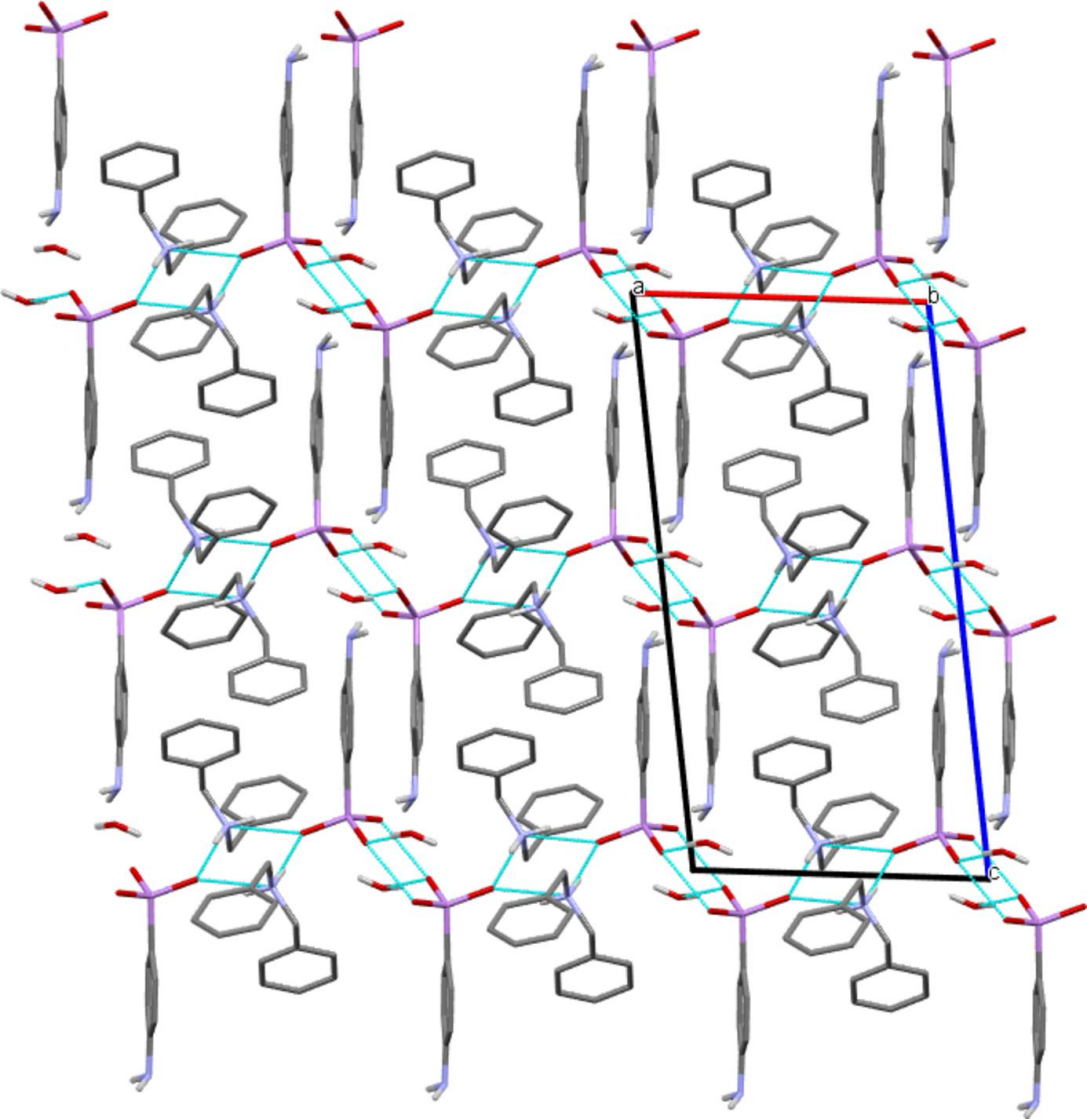
Arrangement of the chains in the crystal of **I** and along the *b*-axis, leading to a three-dimensional network (Mercury representation; Macrae *et al.*, 2020 ▸; colour code: C = grey, N = blue, O = red, As = pink, H = white). H atoms of phenyl and benzyl groups are omitted for clarity. The hydrogen bonds propagating the infinite chains are represented by dotted cyan lines.

**Figure 5:**
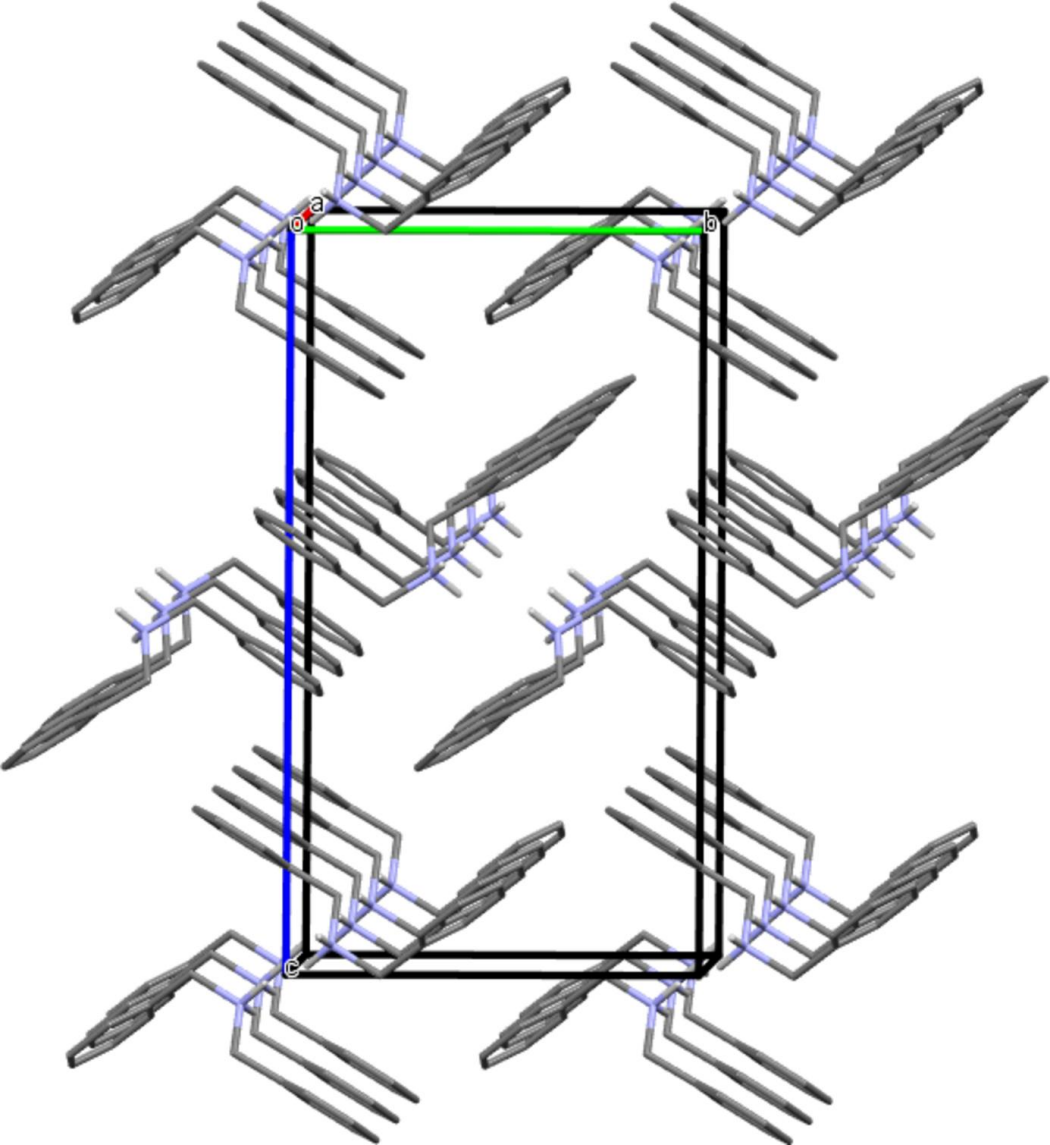
View of the π–π stacking inter­actions between phenyl rings of the di­benzyl­ammonium cations of **I** [along the *a*-axis, Mercury representation (Macrae *et al.*, 2020 ▸); colour code: C = grey, N = blue, H = white). H atoms of phenyl rings, anions and water mol­ecules have been omitted for clarity.

At the supra­molecular stage, two levels of organization can be observed in the crystal structure of **I**:

(i) The propagation of one-dimensional zigzag chains along the *a*-axis direction resulting from the hydrogen-bonding inter­actions (Fig. 2 ▸). The NH_2_ groups of two di­benzyl­ammonium cations are involved in two independent hydrogen bonds, oriented perpendicularly [O3⋯N1⋯O3 = 92.63 (5)°], with the oxygen atoms O3 of two arsonate moieties [N1—H1*A*⋯O3 and N1—H1*B*⋯O3^iii^, Table 1 ▸]. This leads to the formation of a tetra­meric unit describing a four-membered ring (Fig. 3 ▸). These units are linked together by two additional and parallel hydrogen bonds involving two hydrogen (4-amino­phen­yl)arsonate anions [O1—H1⋯O2^iv^, Table 1 ▸]. This creates a six-membered ring. In addition, the water mol­ecule contained in **I** is also in hydrogen-bonding inter­action with the oxygen atom O2 of the arsonate group [O4—H4*A*⋯O2^v^, Table 1 ▸]. The 4-amino­phenyl groups can be viewed as perpendicular to the chain axis and positioned alternately on either side of it.

(ii) The association of chains leading to a three-dimensional network and resulting from a combination of weak inter­actions (Fig. 4 ▸). Two types of π–π stacking inter­actions involving the phenyl rings of the di­benzyl­ammonium cations can be described (Fig. 5 ▸): (*a*) centroid(C15–C20)–centroid (C15^i^–C20^i^) = 3.9384 (10) Å, inter­planar distance = 3.4310 (18) Å, slip angle (angle between the normal to the plane and the centroid–centroid vector) = 29.4, corresponding to a slippage distance of 1.933 Å; symmetry code: (i) 1 − *x*, 2 − *y*, 1 − *z*; (*b*) centroid(C8–C13)–centroid(C15^ii^–C20^ii^) = 4.0178 (10) Å, inter­planar distance = 3.5093 (6) Å, slip angle = 29.1°, corresponding to a slippage distance of 1.957 Å; symmetry code: (ii) 1 − *x*, − 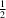 + *y*, 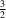 − *z*. In addition, the NH_2_ groups located in the *para* position of C_6_H_4_As(OH)O_2_, inter­act *via* hydrogen bonding with a water mol­ecule [N2—H2*A*⋯O4^1^ = 3.165 (2) Å] and the O1 oxygen atom of an adjacent –As(OH)O_2_ function [N2—H2*B*⋯O1^ii^ = 3.0769 (17) Å] (symmetry codes as in Table 1 ▸).

## 4. Database survey

A search of the Cambridge Structural Database (WebCSD update 11/2022; Groom *et al.*, 2016 ▸), revealed that, to date, there are relatively few X-ray structures exhibiting the isolated hydrogen phenyl­arsonate moiety, C_6_H_5_As(OH)O_2_^−^. To our knowledge, eleven examples including this fragment have already been identified: ammonium 4-nitro­phenyl­arsonate (AHILAE: Yang *et al.*, 2002 ▸), guanidinium phenyl­arsonate guanidine dihydrate (DUSCIE: Smith & Wermuth, 2010 ▸), *p*-amino­phenyl­arsonic acid (CUDSEZ: Shimada, 1961 ▸; CUDSEZ01: Nuttall & Hunter, 1996 ▸), ammonium hydrogen (4-amino­phen­yl)arsonate monohydrate (KOKWOY: Smith & Wermuth, 2014 ▸), 1-(4-hy­droxy-2-methyl­phen­yl)-2,4,6-tri­phenyl­pyridinium hydrogen *o*-arsanilate monohydrate (PAZRIS: Wojtas *et al.*, 2006 ▸), tetra­butyl­ammonium hydrogen phenyl­arsonate–phenyl­arsonic acid (QECBEH: Reck & Schmitt, 2012 ▸), 3-ammonio-4-hy­droxy­phenyl­arsonate (ROBDAO: Lloyd *et al.*, 2008 ▸), hexa­aqua­manganese(II) bis­[hydrogen (4-amino­phen­yl)arsonate] tetra­hydrate (UBURIV: Smith & Wermuth, 2016*a* ▸), hexa­aqua-magnesium bis­(hydrogen (4-amino­phen­yl)arsonate) tetra­hydrate (UDAPIB: Smith & Wermuth, 2017*a* ▸), 2,3-dimeth­oxy-10-oxostrychnidin-19-ium hydrogen (4-amino­phen­yl)arsonate tetra­hydrate (ULIROY: Smith & Wermuth, 2016*b* ▸), 2,4-di­amino-5-(3,4,5-tri­meth­oxy­benz­yl)pyrimidinium 4-hy­droxy-3-nitro­phenyl­arsonate monohydrate (XEMZIZ: Pan *et al.*, 2006 ▸). In coordination chemistry, phenyl­arsonic acid and its derivatives constitute also suitable ligands to generate coordination polymers and heteropolyoxometalates in the presence of transition metals (Lesikar-Parrish *et al.*, 2013 ▸), main-group metals (Xie *et al.*, 2008 ▸), alkali metals (Smith & Wermuth, 2017*a* ▸) and alkali-earth metal precursors (Smith & Wermuth, 2017*b* ▸). Regarding the di­benzyl­ammonium cation, [(C_6_H_5_CH_2_)_2_NH_2_]^+^, 117 hits incorporating such an entity were found in the Cambridge Structural Database.

## 5. Synthesis and crystallization

All chemicals were purchased from Sigma-Aldrich (Germany) and used without any further purification. (4-Amino­phen­yl)arsonic acid [H_2_NC_6_H_4_As(OH)_2_O] was prepared according to a previous work (Lewis & Cheetham, 1923 ▸), by reacting aniline (C_6_H_5_NH_2_) and arsenic acid (As(OH)_3_O). The title salt was obtained by neutralization of an aqueous solution (20 mL) of (4-amino­phen­yl)arsonic acid (2.15 g, 9.90 mmol) with di­benzyl­amine ((C_6_H_5_CH_2_)_2_NH) (3.90 g, 19.80 mmol) dissolved in 20 mL of ethanol. The mixture was stirred for about two h at room temperature (301 K). After three days of slow solvent evaporation, colourless prism-shaped crystals of [(C_6_H_5_CH_2_)_2_NH_2_][H_2_NC_6_H_4_As(OH)O_2_]·H_2_O (5.25 g, 64% yield), suitable for an X-ray crystallographic analysis, were collected from the solvent (m.p. 393 K). FT–IR (ATR, Bruker Alpha FTIR spectrometer, cm^−1^): 3447, 3304, 3187, 1595, 1501, 1454, 1096, 923, 878, 825,752, 735, 695. Elemental analysis (Elemental Analyser, ThermoFisher FlashSmart CHNS/O) – analysis calculated for C_20_H_23_N_2_O_3_As·0.25H_2_O (418.83), salt **I** partially dehydrated: C, 57.35; H, 5.66; N, 6.69; O, 12.41; found: C, 57.82; H, 5.61; N, 6.62; O, 12.37%.

## 6. Refinement details

Crystal data, data collection and structure refinement details are summarized in Table 2 ▸. The asymmetric unit contains the di­benzyl­ammonium hydrogen (4-amino­phen­yl)arsonate mono­hydrate. The water mol­ecule was found disordered over two main positions with occupancy factors that converged to 0.94:0.06. Hence, the minor part of the water mol­ecule was refined only isotropically and without the hydrogen atoms. The hydrogen atoms for the major component of the water mol­ecule were refined geometrically as a rigid group (O—H = 0.87 Å) with *U*_iso_(H) = 1.5*U*_eq_(O). C-bound hydrogen atoms were placed at calculated positions [C—H = 0.95 Å (aromatic) or 0.99 Å (methyl­ene group)] and H atoms of the NH_2_ and OH terminal groups were placed geometrically (N—H = 0.83–0.84 Å, O—H = 0.83 Å) and refined as riding with *U*_iso_(H) = 1.2*U*_eq_(N, C).

## Supplementary Material

Crystal structure: contains datablock(s) I, global. DOI: 10.1107/S205698902300837X/dj2065sup1.cif

Structure factors: contains datablock(s) I. DOI: 10.1107/S205698902300837X/dj2065Isup2.hkl

Supporting information file. DOI: 10.1107/S205698902300837X/dj2065Isup3.cml

CCDC reference: 2297206

Additional supporting information:  crystallographic information; 3D view; checkCIF report

## Figures and Tables

**Table 1:** Hydrogen-bond geometry (Å, °)

*D*—H⋯*A*	*D*—H	H⋯*A*	*D*⋯*A*	*D*—H⋯*A*
N2—H2*A*⋯O4^i^	0.84 (2)	2.37 (2)	3.165 (2)	158.0 (18)
N2—H2*B*⋯O1^ii^	0.83 (2)	2.25 (2)	3.0769 (17)	175.6 (18)
N1—H1*A*⋯O3	0.91	1.78	2.6842 (16)	172
N1—H1*B*⋯O3^iii^	0.91	1.89	2.7260 (15)	151
O1—H1⋯O2^iv^	0.83 (3)	1.73 (3)	2.5445 (15)	170 (3)
O4—H4*A*⋯O2^v^	0.87	1.95	2.8074 (18)	169

Symmetry codes: (i) 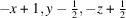 ; (ii) 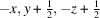 ; (iii) 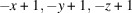 ; (iv) 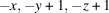 ; (v) 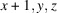 .

**Table 2:** Experimental details

Crystal data	
Chemical formula	C_14_H_16_N^+^·C_6_H_7_AsNO_3_^−^·H_2_O
*M*_r_	432.34
Crystal system, space group	Monoclinic, *P*2_1_/*c*
Temperature (K)	100
*a*, *b*, *c* (Å)	9.8242 (5), 10.6574 (6), 19.2507 (11)
β (°)	97.7500 (18)
*V* (Å^3^)	1997.15 (19)
*Z*	4
Radiation type	Mo *K*α
μ (mm^−1^)	1.73
Crystal size (mm)	0.5 × 0.25 × 0.18

Data collection	
Diffractometer	Bruker D8 VENTURE
Absorption correction	Multi-scan (*SADABS*; Krause *et al.*, 2015 ▸)
*T*_min_, *T*_max_	0.610, 0.746
No. of measured, independent and observed [*I* > 2σ(*I*)] reflections	67932, 4584, 4119
*R*_int_	0.037
(sin θ/λ)_max_ (Å^−1^)	0.650

Refinement	
*R*[*F* ^2^ > 2σ(*F* ^2^)], *wR*(*F* ^2^), *S*	0.022, 0.054, 1.07
No. of reflections	4584
No. of parameters	261
H-atom treatment	H atoms treated by a mixture of independent and constrained refinement
Δρ_max_, Δρ_min_ (e Å^−3^)	0.42, −0.21

Computer programs: *APEX2* (Bruker, 2014 ▸), *SAINT* (Bruker, 2013 ▸), *SHELXT* (Sheldrick, 2015*a* ▸), *SHELXL* (Sheldrick, 2015*b* ▸) and *OLEX2* (Dolomanov *et al.*, 2009 ▸).

## supplementary crystallographic information

### Crystal data

**Table:**

C_14_H_16_N^+^·C_6_H_7_AsNO_3_^−^·H_2_O	*F*(000) = 896
*M**_r_* = 432.34	*D*_x_ = 1.438 Mg m^−^^3^
Monoclinic, *P*2_1_/*c*	Mo *K*α radiation, λ = 0.71073 Å
*a* = 9.8242 (5) Å	Cell parameters from 9824 reflections
*b* = 10.6574 (6) Å	θ = 2.8–27.5°
*c* = 19.2507 (11) Å	µ = 1.73 mm^−^^1^
β = 97.7500 (18)°	*T* = 100 K
*V* = 1997.15 (19) Å^3^	Prism, clear light colourless
*Z* = 4	0.5 × 0.25 × 0.18 mm

### Data collection

**Table:**

Bruker D8 VENTURE diffractometer	4584 independent reflections
Radiation source: X-ray tube, Siemens KFF Mo 2K-90C	4119 reflections with *I* > 2σ(*I*)
TRIUMPH curved crystal monochromator	*R*_int_ = 0.037
Detector resolution: 1024 x 1024 pixels mm^-1^	θ_max_ = 27.5°, θ_min_ = 2.8°
φ and ω scans	*h* = −12→12
Absorption correction: multi-scan (SADABS; Krause *et al.*, 2015)	*k* = −13→13
*T*_min_ = 0.610, *T*_max_ = 0.746	*l* = −24→25
67932 measured reflections	

### Refinement

**Table:**

Refinement on *F*^2^	Primary atom site location: dual
Least-squares matrix: full	Hydrogen site location: mixed
*R*[*F*^2^ > 2σ(*F*^2^)] = 0.022	H atoms treated by a mixture of independent and constrained refinement
*wR*(*F*^2^) = 0.054	*w* = 1/[σ^2^(*F*_o_^2^) + (0.0228*P*)^2^ + 1.3431*P*] where *P* = (*F*_o_^2^ + 2*F*_c_^2^)/3
*S* = 1.07	(Δ/σ)_max_ = 0.001
4584 reflections	Δρ_max_ = 0.42 e Å^−^^3^
261 parameters	Δρ_min_ = −0.21 e Å^−^^3^
0 restraints	

### Special details

**Table:**

Geometry. All esds (except the esd in the dihedral angle between two l.s. planes) are estimated using the full covariance matrix. The cell esds are taken into account individually in the estimation of esds in distances, angles and torsion angles; correlations between esds in cell parameters are only used when they are defined by crystal symmetry. An approximate (isotropic) treatment of cell esds is used for estimating esds involving l.s. planes.

### Fractional atomic coordinates and isotropic or equivalent isotropic displacement parameters (Å^2^)

**Table:**

	*x*	*y*	*z*	*U*_iso_*/*U*_eq_	Occ. (<1)
As	0.15165 (2)	0.51188 (2)	0.42563 (2)	0.01186 (5)	
O1	0.04372 (11)	0.38487 (9)	0.42927 (6)	0.0184 (2)	
O2	0.09995 (10)	0.63317 (9)	0.47065 (5)	0.0167 (2)	
O3	0.31128 (10)	0.46779 (10)	0.45696 (5)	0.0176 (2)	
N2	0.08041 (14)	0.62660 (14)	0.11336 (7)	0.0208 (3)	
H2A	0.062 (2)	0.565 (2)	0.0867 (10)	0.025*	
H2B	0.043 (2)	0.694 (2)	0.1009 (10)	0.025*	
C1	0.13469 (13)	0.55046 (13)	0.32872 (7)	0.0126 (3)	
C2	0.13667 (14)	0.45347 (13)	0.27999 (7)	0.0152 (3)	
H2	0.149937	0.369445	0.295952	0.018*	
C3	0.11956 (15)	0.47831 (14)	0.20880 (7)	0.0164 (3)	
H3	0.121018	0.411363	0.176326	0.020*	
C4	0.10001 (14)	0.60216 (14)	0.18428 (7)	0.0150 (3)	
C5	0.10337 (14)	0.69966 (13)	0.23332 (8)	0.0166 (3)	
H5	0.094757	0.784146	0.217604	0.020*	
C6	0.11920 (14)	0.67391 (13)	0.30476 (7)	0.0150 (3)	
H6	0.119468	0.740721	0.337450	0.018*	
O4	0.94231 (17)	0.85325 (13)	0.45149 (7)	0.0389 (3)	0.94
H4A	0.999317	0.790435	0.455138	0.058*	0.94
H4B	0.870836	0.825432	0.468917	0.058*	0.94
N1	0.43532 (12)	0.63057 (11)	0.55144 (6)	0.0142 (2)	
H1A	0.391629	0.570678	0.522995	0.017*	
H1B	0.523283	0.604491	0.564385	0.017*	
C7	0.36565 (15)	0.64208 (13)	0.61550 (7)	0.0156 (3)	
H7A	0.268703	0.667011	0.601690	0.019*	
H7B	0.411155	0.708549	0.646221	0.019*	
C8	0.37054 (14)	0.52036 (13)	0.65533 (7)	0.0134 (3)	
C9	0.49603 (15)	0.46656 (14)	0.68231 (8)	0.0167 (3)	
H9	0.579319	0.506225	0.674660	0.020*	
C10	0.50021 (15)	0.35571 (14)	0.72019 (8)	0.0194 (3)	
H10	0.586162	0.319539	0.738161	0.023*	
C11	0.37894 (16)	0.29743 (15)	0.73190 (8)	0.0227 (3)	
H11	0.381734	0.221469	0.757878	0.027*	
C12	0.25392 (16)	0.35050 (15)	0.70557 (9)	0.0251 (3)	
H12	0.170863	0.311054	0.713808	0.030*	
C13	0.24933 (15)	0.46135 (15)	0.66711 (8)	0.0200 (3)	
H13	0.163208	0.496855	0.648829	0.024*	
C14	0.43754 (16)	0.75047 (14)	0.51084 (8)	0.0194 (3)	
H14A	0.342130	0.775473	0.493084	0.023*	
H14B	0.486851	0.736507	0.469927	0.023*	
C15	0.50659 (15)	0.85489 (14)	0.55510 (8)	0.0182 (3)	
C16	0.42794 (16)	0.94873 (15)	0.57986 (8)	0.0220 (3)	
H16	0.330863	0.947490	0.568182	0.026*	
C17	0.49027 (19)	1.04468 (15)	0.62169 (9)	0.0277 (4)	
H17	0.435865	1.108628	0.638562	0.033*	
C18	0.6313 (2)	1.04676 (17)	0.63861 (9)	0.0322 (4)	
H18	0.673979	1.112232	0.667157	0.039*	
C19	0.71081 (18)	0.95354 (18)	0.61403 (10)	0.0323 (4)	
H19	0.807876	0.955423	0.625699	0.039*	
C20	0.64919 (16)	0.85748 (16)	0.57247 (9)	0.0249 (3)	
H20	0.703946	0.793541	0.555854	0.030*	
H1	0.002 (3)	0.386 (3)	0.4638 (14)	0.062 (8)*	
O4B	0.729 (2)	0.7346 (18)	0.4434 (10)	0.026 (4)*	0.06

### Atomic displacement parameters (Å^2^)

**Table:**

	*U*^11^	*U*^22^	*U*^33^	*U*^12^	*U*^13^	*U*^23^
As	0.01149 (7)	0.01330 (7)	0.01092 (7)	−0.00011 (5)	0.00195 (5)	−0.00127 (5)
O1	0.0235 (5)	0.0140 (5)	0.0192 (5)	−0.0066 (4)	0.0084 (4)	−0.0043 (4)
O2	0.0200 (5)	0.0125 (5)	0.0187 (5)	−0.0005 (4)	0.0070 (4)	−0.0030 (4)
O3	0.0143 (5)	0.0226 (5)	0.0150 (5)	0.0041 (4)	−0.0009 (4)	−0.0025 (4)
N2	0.0233 (7)	0.0235 (7)	0.0151 (6)	0.0019 (6)	0.0010 (5)	0.0037 (5)
C1	0.0096 (6)	0.0163 (6)	0.0119 (6)	−0.0005 (5)	0.0018 (5)	0.0004 (5)
C2	0.0157 (7)	0.0132 (6)	0.0164 (7)	0.0004 (5)	0.0011 (5)	0.0009 (5)
C3	0.0179 (7)	0.0164 (7)	0.0147 (7)	0.0000 (5)	0.0013 (5)	−0.0022 (5)
C4	0.0097 (6)	0.0198 (7)	0.0158 (7)	0.0003 (5)	0.0026 (5)	0.0029 (5)
C5	0.0149 (7)	0.0140 (6)	0.0211 (7)	0.0016 (5)	0.0035 (5)	0.0042 (5)
C6	0.0127 (6)	0.0147 (6)	0.0179 (7)	−0.0005 (5)	0.0035 (5)	−0.0018 (5)
O4	0.0569 (10)	0.0308 (7)	0.0304 (7)	0.0169 (7)	0.0104 (7)	0.0108 (6)
N1	0.0149 (6)	0.0140 (6)	0.0132 (6)	−0.0003 (4)	0.0007 (4)	0.0000 (4)
C7	0.0170 (7)	0.0144 (6)	0.0160 (7)	0.0009 (5)	0.0045 (5)	−0.0008 (5)
C8	0.0143 (6)	0.0137 (6)	0.0123 (6)	−0.0003 (5)	0.0023 (5)	−0.0020 (5)
C9	0.0129 (6)	0.0194 (7)	0.0181 (7)	−0.0015 (5)	0.0031 (5)	0.0003 (6)
C10	0.0160 (7)	0.0223 (7)	0.0198 (7)	0.0047 (6)	0.0016 (6)	0.0031 (6)
C11	0.0253 (8)	0.0182 (7)	0.0247 (8)	0.0005 (6)	0.0043 (6)	0.0059 (6)
C12	0.0172 (7)	0.0229 (8)	0.0354 (9)	−0.0053 (6)	0.0044 (7)	0.0068 (7)
C13	0.0119 (7)	0.0214 (7)	0.0260 (8)	−0.0005 (5)	−0.0001 (6)	0.0028 (6)
C14	0.0230 (8)	0.0187 (7)	0.0161 (7)	−0.0018 (6)	0.0010 (6)	0.0048 (6)
C15	0.0204 (7)	0.0173 (7)	0.0169 (7)	−0.0042 (6)	0.0022 (6)	0.0057 (6)
C16	0.0226 (8)	0.0191 (7)	0.0246 (8)	−0.0032 (6)	0.0050 (6)	0.0045 (6)
C17	0.0409 (10)	0.0180 (7)	0.0256 (8)	−0.0041 (7)	0.0095 (7)	0.0018 (6)
C18	0.0434 (10)	0.0244 (8)	0.0276 (9)	−0.0172 (8)	0.0004 (8)	0.0012 (7)
C19	0.0237 (8)	0.0356 (10)	0.0358 (10)	−0.0118 (7)	−0.0023 (7)	0.0051 (8)
C20	0.0207 (8)	0.0257 (8)	0.0283 (8)	−0.0022 (6)	0.0037 (6)	0.0044 (7)

### Geometric parameters (Å, º)

**Table:**

As—O1	1.7267 (10)	C7—C8	1.5044 (19)
As—O2	1.6730 (10)	C8—C9	1.395 (2)
As—O3	1.6699 (10)	C8—C13	1.392 (2)
As—C1	1.8955 (13)	C9—H9	0.9500
O1—H1	0.83 (3)	C9—C10	1.386 (2)
N2—H2A	0.84 (2)	C10—H10	0.9500
N2—H2B	0.83 (2)	C10—C11	1.389 (2)
N2—C4	1.3776 (19)	C11—H11	0.9500
C1—C2	1.3978 (19)	C11—C12	1.385 (2)
C1—C6	1.3957 (19)	C12—H12	0.9500
C2—H2	0.9500	C12—C13	1.392 (2)
C2—C3	1.384 (2)	C13—H13	0.9500
C3—H3	0.9500	C14—H14A	0.9900
C3—C4	1.406 (2)	C14—H14B	0.9900
C4—C5	1.401 (2)	C14—C15	1.506 (2)
C5—H5	0.9500	C15—C16	1.387 (2)
C5—C6	1.391 (2)	C15—C20	1.396 (2)
C6—H6	0.9500	C16—H16	0.9500
O4—H4A	0.8696	C16—C17	1.391 (2)
O4—H4B	0.8701	C17—H17	0.9500
N1—H1A	0.9100	C17—C18	1.380 (3)
N1—H1B	0.9100	C18—H18	0.9500
N1—C7	1.4939 (17)	C18—C19	1.386 (3)
N1—C14	1.4995 (18)	C19—H19	0.9500
C7—H7A	0.9900	C19—C20	1.387 (2)
C7—H7B	0.9900	C20—H20	0.9500
			
O1—As—C1	103.71 (6)	C13—C8—C7	120.22 (13)
O2—As—O1	110.71 (5)	C13—C8—C9	119.09 (13)
O2—As—C1	110.47 (6)	C8—C9—H9	119.7
O3—As—O1	108.46 (5)	C10—C9—C8	120.55 (13)
O3—As—O2	111.48 (5)	C10—C9—H9	119.7
O3—As—C1	111.73 (5)	C9—C10—H10	120.0
As—O1—H1	113.0 (19)	C9—C10—C11	120.09 (14)
H2A—N2—H2B	116.7 (18)	C11—C10—H10	120.0
C4—N2—H2A	116.7 (13)	C10—C11—H11	120.1
C4—N2—H2B	116.7 (13)	C12—C11—C10	119.73 (14)
C2—C1—As	119.52 (10)	C12—C11—H11	120.1
C6—C1—As	121.39 (10)	C11—C12—H12	119.8
C6—C1—C2	119.08 (13)	C11—C12—C13	120.33 (14)
C1—C2—H2	119.6	C13—C12—H12	119.8
C3—C2—C1	120.83 (13)	C8—C13—H13	119.9
C3—C2—H2	119.6	C12—C13—C8	120.20 (14)
C2—C3—H3	119.8	C12—C13—H13	119.9
C2—C3—C4	120.33 (13)	N1—C14—H14A	109.2
C4—C3—H3	119.8	N1—C14—H14B	109.2
N2—C4—C3	120.31 (13)	N1—C14—C15	111.83 (12)
N2—C4—C5	120.99 (13)	H14A—C14—H14B	107.9
C5—C4—C3	118.69 (13)	C15—C14—H14A	109.2
C4—C5—H5	119.7	C15—C14—H14B	109.2
C6—C5—C4	120.62 (13)	C16—C15—C14	119.86 (14)
C6—C5—H5	119.7	C16—C15—C20	119.40 (14)
C1—C6—H6	119.8	C20—C15—C14	120.73 (14)
C5—C6—C1	120.37 (13)	C15—C16—H16	119.8
C5—C6—H6	119.8	C15—C16—C17	120.45 (15)
H4A—O4—H4B	104.5	C17—C16—H16	119.8
H1A—N1—H1B	107.7	C16—C17—H17	120.1
C7—N1—H1A	108.8	C18—C17—C16	119.86 (16)
C7—N1—H1B	108.8	C18—C17—H17	120.1
C7—N1—C14	113.62 (11)	C17—C18—H18	119.9
C14—N1—H1A	108.8	C17—C18—C19	120.13 (16)
C14—N1—H1B	108.8	C19—C18—H18	119.9
N1—C7—H7A	109.4	C18—C19—H19	119.9
N1—C7—H7B	109.4	C18—C19—C20	120.25 (16)
N1—C7—C8	111.30 (11)	C20—C19—H19	119.9
H7A—C7—H7B	108.0	C15—C20—H20	120.0
C8—C7—H7A	109.4	C19—C20—C15	119.91 (16)
C8—C7—H7B	109.4	C19—C20—H20	120.0
C9—C8—C7	120.67 (13)		
			
As—C1—C2—C3	177.66 (11)	N1—C14—C15—C20	74.49 (17)
As—C1—C6—C5	−178.33 (10)	C7—N1—C14—C15	57.65 (16)
O1—As—C1—C2	−44.30 (12)	C7—C8—C9—C10	178.70 (13)
O1—As—C1—C6	135.23 (11)	C7—C8—C13—C12	−178.27 (14)
O2—As—C1—C2	−162.96 (10)	C8—C9—C10—C11	−0.3 (2)
O2—As—C1—C6	16.57 (13)	C9—C8—C13—C12	0.3 (2)
O3—As—C1—C2	72.32 (12)	C9—C10—C11—C12	0.0 (2)
O3—As—C1—C6	−108.15 (11)	C10—C11—C12—C13	0.4 (3)
N2—C4—C5—C6	178.11 (13)	C11—C12—C13—C8	−0.6 (2)
C1—C2—C3—C4	0.1 (2)	C13—C8—C9—C10	0.2 (2)
C2—C1—C6—C5	1.2 (2)	C14—N1—C7—C8	−178.14 (11)
C2—C3—C4—N2	−178.78 (13)	C14—C15—C16—C17	179.01 (14)
C2—C3—C4—C5	2.3 (2)	C14—C15—C20—C19	−179.17 (15)
C3—C4—C5—C6	−3.0 (2)	C15—C16—C17—C18	0.1 (2)
C4—C5—C6—C1	1.3 (2)	C16—C15—C20—C19	−0.1 (2)
C6—C1—C2—C3	−1.9 (2)	C16—C17—C18—C19	0.0 (3)
N1—C7—C8—C9	60.72 (17)	C17—C18—C19—C20	−0.2 (3)
N1—C7—C8—C13	−120.76 (14)	C18—C19—C20—C15	0.2 (3)
N1—C14—C15—C16	−104.55 (16)	C20—C15—C16—C17	0.0 (2)

### Hydrogen-bond geometry (Å, º)

**Table:**

*D*—H···*A*	*D*—H	H···*A*	*D*···*A*	*D*—H···*A*
N2—H2*A*···O4^i^	0.84 (2)	2.37 (2)	3.165 (2)	158.0 (18)
N2—H2*B*···O1^ii^	0.83 (2)	2.25 (2)	3.0769 (17)	175.6 (18)
N1—H1*A*···O3	0.91	1.78	2.6842 (16)	172
N1—H1*B*···O3^iii^	0.91	1.89	2.7260 (15)	151
O1—H1···O2^iv^	0.83 (3)	1.73 (3)	2.5445 (15)	170 (3)
O4—H4*A*···O2^v^	0.87	1.95	2.8074 (18)	169

Symmetry codes: (i) −*x*+1, *y*−1/2, −*z*+1/2; (ii) −*x*, *y*+1/2, −*z*+1/2; (iii) −*x*+1, −*y*+1, −*z*+1; (iv) −*x*, −*y*+1, −*z*+1; (v) *x*+1, *y*, *z*.
